# Appetitive Aggression in Women: Comparing Male and Female War Combatants

**DOI:** 10.3389/fpsyg.2015.01972

**Published:** 2016-01-05

**Authors:** Danie Meyer-Parlapanis, Roland Weierstall, Corina Nandi, Manassé Bambonyé, Thomas Elbert, Anselm Crombach

**Affiliations:** ^1^Department of Psychology, University of KonstanzKonstanz, Germany; ^2^Department of Clinical Psychology, Université LumièreBujumbura, Burundi

**Keywords:** appetitive aggression, sex differences, female combatants, armed conflicts, Burundi, female perpetrators, female aggression, combat stress

## Abstract

Appetitive aggression refers to positive feelings being associated with the perpetration of violent behavior and has been shown to provide resilience against the development of PTSD in combatants returning from the battlefield. Until this point, appetitive aggression has been primarily researched in males. This study investigates appetitive aggression in females. Female and male combatants and civilians from Burundi were assessed for levels of appetitive aggression. In contrast to non-combatants, no sex difference in appetitive aggression could be detected for combatants. Furthermore, each of the female and male combatant groups displayed substantially higher levels of appetitive aggression than each of the male and female civilian control groups. This study demonstrates that in violent contexts, such as armed conflict, in which individuals perpetrate numerous aggressive acts against others, the likelihood for an experience of appetitive aggression increases- regardless of whether the individuals are male or female.

## Introduction

Following armed conflict and war, each affected society faces a myriad of challenges to efforts at healing and rebuilding. Considering the psychological well-being of the individuals impacted by war, the participating combatants were often directly and repeatedly affected by the violence due to their participation on the front lines. Combatants have a complex experience of war that simultaneously incorporates surviving and attacking. In each individual occurrence of violence, combatants must juggle the roles between operating as perpetrators and survivors (Schauer and Elbert, 2010; Meyer-Parlapanis and Elbert, 2015).

As survivors, combatants must navigate both the battlefield and the mosaic of other possible threats that exist during wartime (i.e., limited food or water, natural disasters, violence from compatriots, etc.). These numerous experiences in which their own lives, their loved ones and resources are threatened can induce negative emotions and be potentially traumatizing. When a combatant predominantly experiences negative emotions associated with the violence of combat, the likelihood for the development of Post-Traumatic Stress Disorder (PTSD) increases (Neuner et al., 2004; Hecker et al., 2013a). PTSD is also often accompanied by comorbid disorders, such as Major Depression, Dysthymia, Phobias, Alcohol and Drug Abuse, and Conduct Disorder (Kessler et al., 1995), further affecting the mental health of many returning combatants. However, the challenges for former combatants returning from the battlefield are not limited to the mental health complications stemming from their experiences as survivors.

Albeit not currently as clear as the relationship between experiencing numerous potentially traumatic events and PTSD, the experience of perpetrating violence is being shown to play an additional and significant role in the well-being of combatants following war (Köbach et al., 2014). Aggressive behavior during combat usually is a mixture of reactive aggression (an impulsive, affective and uncontrolled violent behavior provoked by a perceived or real threat) and appetitive aggression motivated by intrinsic reward, describing the human potential to perceive perpetrated violence as fascinating and exciting (Elbert et al., 2010). The current understanding of appetitive aggression is based on the testimonies of thousands of combatants reporting feelings of excitement and fascination at committing violent acts (MacNair, 2006; Weierstall and Elbert, 2011; Weierstall et al., 2011, 2013; Crombach et al., 2013). Several studies indicate that appetitive aggression might be an adaptation to cruel and violent environments, such as battlefields. Apparently, combatants who developed high levels of appetitive aggression remain more functional in such settings, gain an elevated social status within armed forces and adapt better to their violent tasks thereby increasing their ability to prevail (Crombach et al., 2013). These particular impacts of appetitive aggression are not isolated to the battlefield. Combatants reporting positive emotions in association with violent combat, appetitive aggression, have also been found to be at lower risk for the development of PTSD (Weierstall et al., 2011, 2012a,b; Hecker et al., 2013b). The different quality of appetitive aggression as opposed to reactive aggression has also been validated by a difference in associated neural systems (Moran et al., 2014). The experience of appetitive aggression can complicate the transition of former combatants back to civilian life. For example, some individuals may feel the need to perpetrate further violence in an effort to replicate the rewarding experience, i.e., higher levels of appetitive aggression have been related to higher rates of reenlistment (Hermenau et al., 2013a). More research on the experience of appetitive aggression is needed to better understand the mental health state and trajectory of combatants following armed conflict.

Investigating appetitive aggression assists in identifying at-risk individuals, sheds light on the complex experience of being a combatant and provides insight for treatment options (Hermenau et al., 2013b; Crombach and Elbert, 2015). Such steps forward in research hopefully translate into a direct increase in mental health knowledge and resources for all combatants returning from the numerous, ongoing armed conflicts around the globe. However, there is a portion of combatants that have often been overlooked. Despite the importance of researching appetitive aggression in former combatants, a literature search revealed no current studies assessing the psychological consequences of perpetrating high levels of violence that include a focus on the female experience.

This oversight is not new. Studies on human aggressive behavior have frequently neglected to include females in the same ratios as males or have failed to include females altogether (Frodi et al., 1977). Moreover, appetitive aggression investigations “have focused almost exclusively on male populations heavily involved in the perpetration of violence” (Crombach and Elbert, 2014, p. 1042). This oversight is possibly due to a general assumption that females are less aggressive than males (Richardson, 2005; Stockley and Campbell, 2013). This assumption is likely grounded in evolutionary theory considering males as more likely to implement aggressive strategies in the competition for social status, wealth, and sexual partners (Wilson et al., 2002). However, this assumption only holds true under certain conditions (Archer, 2004; Campbell, 2006) and neglects the wide variety of aggressive strategies. For example, Campbell (2013) argues that, due to their evolutionary higher involvement in raising children and in an effort not to jeopardize reproductive success, females prefer strategies with lower risk of injury, such as indirect forms of aggression. More studies investigating female expressions of aggression, especially appetitive aggression, are needed because the current, limited number of studies yield inconsistent results. In a sample of Rwandan genocide perpetrators, females reported lower levels of appetitive aggression than males (Weierstall et al., 2011). In contrast, no gender effect in the prediction of appetitive aggression was revealed in a sample of Columbian ex-combatants (Weierstall et al., 2013). This is highly problematic for female combatants dealing with the fallout of psychological complications following war.

In designing a project to best investigate appetitive aggression in male and female combatants, scenarios involving the sensorial elements that can motivate appetitive aggression offer an ideal opportunity. The sights and smells of blood and sweat, the sounds of the battlefield, and the physical sensation of a weapon hitting a target are all sensorial elements that play a vital role in our understanding of the neurological reward systems that assist in the pleasurable sensation involved in appetitive aggression (for details, see e.g., Elbert et al., 2010; Moran et al., 2014; Crombach and Elbert, 2015). Taking these elements into account, Burundi was identified as a country that has endured the type of violence most applicable for this investigation of appetitive aggression in combatants.

Situated in Eastern Africa, Burundi is a country that has endured extreme violence over the last several decades. Ethnic violence between Hutu and Tutsi factions, escalating for more than 40 years, culminated in a civil war in 1993. This war has left more than 300,000 dead, 500,000 refugees, and 800,000 internally displaced Burundians (Uvin, 2009). The fighting style utilized in the war has not employed distant, abstract forms of violence, like programming drones or throwing bombs from an airplane. Rather there were most often battlefronts of two opposing armed groups exchanging fire at mid or close range. In many cases fighting scenarios included close, hand-to-hand assault and torture that produced multi-sensory feedback. Combatants often saw with the naked eye their targets' bodies hit by bullets, felt the impact of their knives and sticks on their targets' bodies, saw the blood pour out of wounds, smelled the sweat and blood of their victims and heard their cries of pain and pleas for mercy, the sensorial elements required for investigating appetitive aggression.

Furthermore, both male and female combatants participated in the violence. Although male combatants predominantly constituted both government and rebel forces, females were also involved in armed forces. Many of the females were originally recruited for such roles as cooks, porters, and messengers and also for sexual purposes or forced marriage (Schauer and Elbert, 2010). However, some females additionally participated in combat and other forms of direct violence, gradually advancing in the military hierarchy of their respective groups. Once having achieved the ranks applicable for fighting on the front lines, the violence experienced and perpetrated during fighting was similar for all combatants on the battlefield, both male and female.

The current investigation explores the experience of appetitive aggression in female and male combatants in Burundi. Acknowledging both the survivor and perpetrator aspects of performing as a combatant in armed conflict, we investigated self-experienced and witnessed traumatic events alongside the aggressive acts perpetrated against others during armed conflict. Particular attention was paid to the current perception of perpetrated acts, distinguishing between acts committed with neutral or negative emotions and those committed with pleasure. Thereby, we investigated whether, like the male combatants from the aforementioned investigations, female combatants can experience aggressive behavior as a fascinating or satisfying endeavor.

## Methods

### Participants

For the overarching investigation, 429 participants (412 males, 17 females) were recruited from a military and a rebel veteran's organization in Bujumbura, Burundi. Of the 17 female participants, 2 participants were excluded due discrepancies in the provided information, leaving 15 female former combatants. Taken from the larger male sample, 15 male former combatants were matched to the 15 female former combatants on the criteria of age, cumulative exposure to traumatic stressors as measured by the number of experienced traumatic event types, offense load as measured by the number of perpetrated event types and current PTSD symptom severity. For a control population, 20 male and 20 female non-combatants were recruited as a random sample from the community and interviewed using the same materials as the combatant group. Equal distribution between the experimental and control groups would have been preferred, however there was greater accessibility to males than to females. Thereby, the decision was made to include as many females as were available rather than artificially limiting the number of participants in their respective groups in an effort to achieve equal distribution.

### Procedure

#### Recruitment

The interviews took place on the Université Lumière Bujumbura campus from July to September 2012 and at the Red Cross Burundi in Gatumba in January 2013. Five psychologists (3 male, 2 female) from the University of Konstanz, with the assistance of local interpreters (all male), conducted the assessments. One local female psychologist from Université Lumière also conducted interviews in Kirundi. To guarantee precise translation, different interpreters translated and back translated all instruments from validated English versions to Kirundi. The translated instruments were discussed in detail with the interpreters before the beginning of data collection.

Furthermore, topic sensitivity and participant safety were covered at length amongst the interviewers before the assessment period began. The interpreters were provided sensitivity training for assisting in interviews with female participants. Such training covered topics related to maximizing the female participants' sense of safety in the interview (e.g., seating arrangements, non-intimidating bodily posturing, averted eye contact, avoiding all physical contact). Additionally, the interpreters had been trained in the relevant concepts of mental disorders and translated between Kirundi, French and English. The interviews averaged 2.5 h in length.

The Ethical Review Boards at the University of Konstanz and the Université Lumière approved the study, and the Université Lumière supported the project by providing workspace in which the interviews could be performed. Participation in the study was voluntary and all participants provided informed consent via signature prior to the interview. Options for verbal informed consent and/or acceptance of the participant's personal symbol were given in cases of illiteracy. All participants gave informed consent and received 10,000 fbu (approximately 5€) as compensation for participation in the study. Participants were recruited through the use of a local recruiter associated with the Veteran's Association in Burundi. With the upmost respect to the vulnerability of the population, secure data encryption via electronic participant coding and password protected storage ensured anonymity and confidentiality.

### Measures

#### Traumatic event types

Participants were assessed for 19 potentially traumatic, life threatening, event types, both witnessed and self-experienced, stemming from war and non-war related events. The checklist was an adapted version of a checklist that has been used previously with populations affected by violent conflicts (Neuner et al., 2004; Nandi et al., 2015), which incorporated all events from the Posttraumatic Stress Diagnostic Scale (Foa et al., 1997) and was adapted to the Burundian cultural context. The frequency of potentially traumatic events was not assessed, due to the lack of reliability associated with memory biases (Kolassa et al., 2010; Wilker et al., 2015). The items were coded dichotomously, with “1” and “0” respectively representing having and not having experienced the event in reference. The sum number of experienced, potentially traumatic event types represents the trauma load.

#### Perpetrated event types

To measure self-committed violence, we systematically assessed 14 different types of perpetrated violence (e.g., mutilation, rape, or killing) with a checklist that has already been utilized in multiple combatant populations (Weierstall and Elbert, 2011). The items were coded dichotomously in the same manner as the traumatic event types and also summed up to represent a self-committed violence sum score. The traumatic and perpetrated event type items can be found in the appendix.

#### PTSD symptom severity

PTSD symptom severity was assessed using the PTSD Symptom Scale-Interview (PSS-I; Foa and Tolin, 2000). The PSS-I is a semi-structured interview based on the 17 DSM-IV (American Psychiatric Association, 2000) symptom criteria for PTSD and measures symptom intensity during the previous month. The PSS-I has established validity in comparable East-African samples (Ertl et al., 2010). PTSD severity was calculated by totaling symptom scores (scores range from 0 to 51). Internal consistency for the PSS-I in the current investigation is appropriate (Cronbach's α = 0.88).

#### Appetitive aggression

Appetitive aggression was assessed using the Appetitive Aggression Scale (AAS; Weierstall and Elbert, 2011). The AAS has been successfully implemented (Hecker et al., 2012; Weierstall et al., 2012b) and validated (Weierstall and Elbert, 2011) in comparable East African samples. The AAS consists of 15 items, which are rated by responses on a five-point scale ranging from 0 (*I totally disagree*) to 4 (*I totally agree*). The items gather information about participants' perception of violence (e.g., “Is it exciting for if you make an opponent really suffer?”; “Once fighting has started, do you get carried away by the violence?”). The AAS score is calculated by adding the scores of the 15 items (scores range from 0 to 60). Psychometric property measures indicated excellent internal consistency (Cronbach's α = 0.94) in the current study.

### Data analysis

In order to investigate potential sex differences in AAS, the 15 female combatant participants were matched to 15 of the 412 male combatant participants prior to comparing AAS sum scores between the sexes. The matches were performed based on 4 factors: age, the sum of experienced traumatic event types, the sum of perpetrated event types, and the sum PSS-I scores as a measure of PTSD symptom severity at the time of the interview. These 4 factors were selected as match criteria in order to ensure that the compared participants would be as similar as possible in the categories that potentially have an influence on appetitive aggression.

The number of experienced traumatic event types and the sum PSS-I scores were selected because of the “building block effect” of traumatic events on trauma symptom severity, i.e., with each traumatic event experienced, the likelihood of developing PTSD increases (Neuner et al., 2004). Considering the mediating role of appetitive aggression in PTSD symptomology, it was imperative to compare individuals who suffered to a similar degree and who had experienced a similar number of traumatic events. The number of perpetrated offense types was selected because, inasmuch as the number of experienced traumatic events has a “building block effect” on trauma symptomology, similar effects with perpetrated acts of violence have been shown (Hecker et al., 2013a,b; Hermenau et al., 2013a). Thereby, individuals having committed a similar number of offenses were compared to one another. Finally, interaction between traumatic experience and committing violent acts has been shown to contribute to appetitive aggression (Nandi et al., 2015), further supporting the decision to control for both traumatic event types and perpetrated event types.

In an effort to reduce error variance, the match method was selected. The pair-wise comparison was completed using the MATCH command-line program created by the Cognitive and Brain Sciences Unit (van Casteren and Davis, 2007). The program performs an optimized, near-exhaustive search through the 9.88 × 10 ^∧^26 possible sets of 15 male combatant participants from the initial set of 412 such that: (1) each female combatant participant was paired with a single male combatant participant that closely matched in age, experienced events types, perpetrated event types and PSS-I scores, (2) each of the 15 male combatant participants was only paired with one female combatant participant, and (3) the summed discrepancy (normalized Euclidean distance) between the pairs of male and female combatant participants on the four critical factors was minimized. A full description of the operation of the program can be found in van Casteren and Davis (2007). Table 1 includes an overview of the aforementioned variables in the matched participant data.

**Table 1:** **Participant demographics and results**.

	**Combatants**		**Non-combatants**	
	**Female (*n* = 15)**	**Male (*n* = 15)**	**Female (*n* = 20)**	**Male (*n* = 20)**
Age, years	27.1	27.6	25.5	26.0
	(1.0)	(1.2)	(1.0)	(1.2)
	[20–32]	[21–35]	[19–33]	[18–38]
Education level	5.8	5.3	5.1	9.9
	(0.9)	(1.0)	(0.5)	(0.6)
	[0–12]	[0–12]	[0–9]	[5–13]
Number of children	2.1	0.5	2.1	0.7
	(0.4)	(0.3)	(3.3)	(0.2)
	[0–6]	[0–3]	[0–4]	[0–3]
Sum traumatic event types	14.3	14.6	10.8	12.1
	(0.7)	(0.4)	(0.6)	(0.7)
	[8–18]	[11–17]	[6–17]	[7–17]
Sum perpetrated event types	9.7	9.9	1.8	2.9
	(1.2)	(1.0)	(0.4)	(0.5)
	[0–15]	[3–14]	[0–6]	[0–8]
PTSD symptom severity	18.3	17.6	16.2	10.8
	(2.7)	(2.7)	(2.0)	(2.0)
	[2–32]	[0–33]	[0–37]	[0–36]

*Mean values of participant data presented. SE appear in parenthesis. Ranges appear in brackets*.

We then assessed the potential group differences between male and female combatants and non-combatants by conducting a Two-way analysis of variance (ANOVA) with the AAS sum score as the dependent variable. The statistical analysis was carried out using SPSS 21 (IBM Corp. Released, 2012).

## Results

Table 1 gives a demographic overview of participant age, year of successful school education and the number of children for female and male combatants and non-combatants. Additionally, the number of traumatic events types, perpetrated event types and PTSD symptom severity are presented. Respective statistical tests confirmed that females had more children than males and that male non-combatants had a higher degree of education (all *p* ≤ 0.015). The load of traumatic event types and PTSD symptom severity was high, even within the non-combatants. Moreover, they did not differ significantly from the combatants regarding the two variables. Overall, the non-combatants perpetrated fewer event types than the combatants (*p* = 0.001). The matched male combatants were found to be on average with the rest of the male combatant population. There was no significant difference in appetitive aggression (AAS_matched_: mean = 34.1, *SE* = 3.7; AAS_non−matched_: mean = 28.4, *SE* = 0.7; *p* = 0.13), PTSD symptom severity (*p* = 0.18), the load of traumatic (*p* = 0.08), and perpetrated event types (*p* = 0.56).

**Figure 1:**
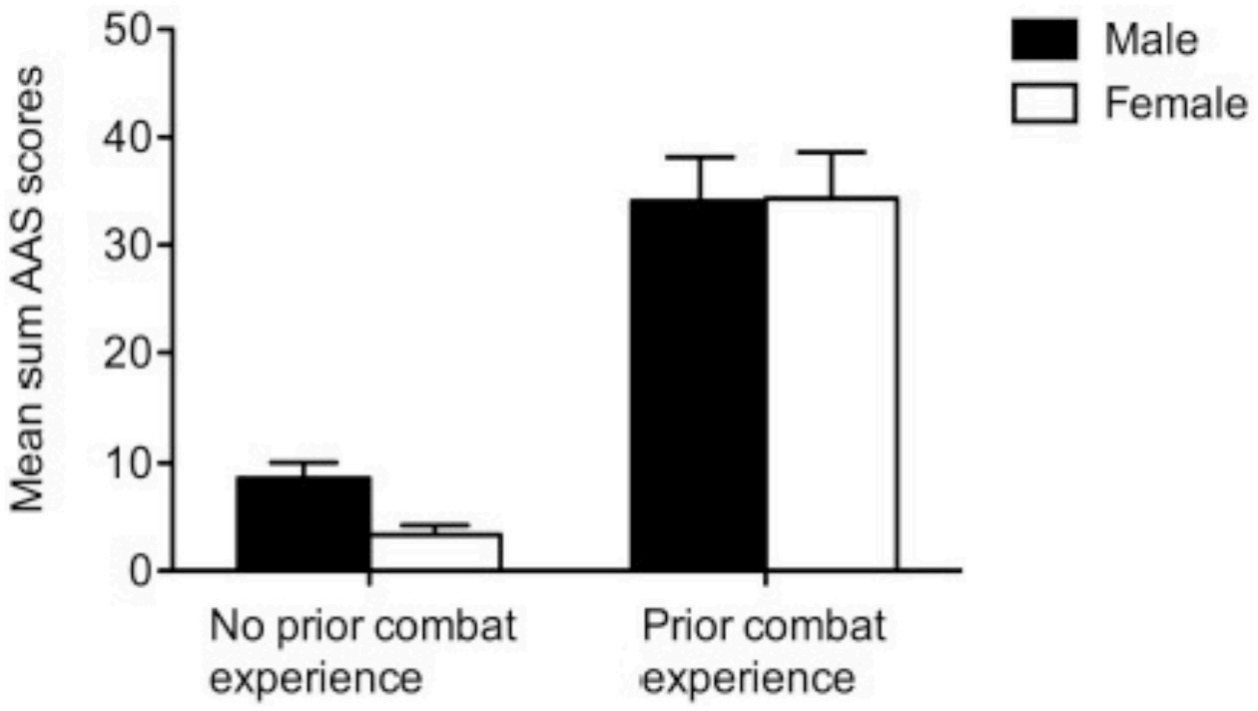
**Mean AAS sum scores in males and females with and without combat experience**. Bars represent standard error.

Normality of the distribution of the AAS scale in each group was verified using Kolmogorov-Smirnov-Tests (all *p* > 0.05) (see Table 2). A Brown–Forsythe robust Levene test of homogeneity of variances based on deviation from the median was calculated (Brown and Forsythe, 1974) and revealed a rejection of the hypothesis of homogeneity of variance [*F*_(3, 66)_ = 5.00, *p* = 0.003]. Comparisons of variances between pairs of groups showed that this effect could be traced back to the variance differences between the two civilian and the two combatant groups [*F*_(1, 66)_ = 14.18; *p* < 0.001], with combatants showing much higher variances. The inequality of the variances across the groups and the relatively small sample size prevented an assumption of robustness of parametric testing. We ensured the robustness of the analyses using non-parametric tests when the data did not match the criteria for parametric testing (Bortz, 2005). Thus, a Kruskal-Wallis test was performed on the AAS scores and revealed statistically significant group differences at *p* < 0.001 (*H* = 38.84, *df* = 3) (see Figure 1). Multiple comparisons between the four groups were conducted by independent sample *t*-tests for comparisons between females and males from the combatant as well as the civilian groups and by Mann-Whitney *U-*tests for comparisons between combatant and civilian groups. The critical *p-*value was Bonferroni adjusted to 0.008. While there was no statistically significant group difference between female and male combatants [*t*_(28)_ = 0.1, *p* = 0.96, *d* = 0.02], male civilians showed statistically significant higher AAS scores than female civilians [*t*_(31, 32)_ = 3.03, *p* = 0.005, *d* = 0.96]. Comparisons between combatants and civilians revealed that both female and male combatants had higher AAS scores than any of the civilian group (all Mann-Whitney *U-*tests < 0.001).

**Table 2:** **Summary of statistics performed on ASS scale**.

	**Non-combatants**		**Combatants**		**Test**	**Comparison**	***t/Z/F***	***p***
	**Female (*n =* 20)**	**Male (*n =* 20)**	**Female (*n =* 20)**	**Male (*n =* 20)**				
ASS score, mean (SD)	3.35 (4.02)	8.60 (6.62)	34.33 (16.56)	34.07 (15.85)	*t*-test^b^	NC female vs. NC male	*t*_(31, 32)_ = 3.03	0.005
						C female vs. C male	*t*_(28)_ = 0.1	0.96
					Mann-Whitney U^b^	NC female vs. C female	*Z* = −4.43	<0.001
						NC female vs. C male	*Z* = −4.45	<0.001
						NC male vs. C female	*Z* = −3.97	<0.001
						NC male vs. C male	*Z* = −4.02	<0.001
AAS score, skewness (SE)	1.22 (0.51)	0.21 (0.51)	−0.54 (0.58)	−0.89 (0.58)	Brown–Forsythe Levene test^c^	Sex	*F*_(1, 66)_ = 0.37	0.545
AAS score, kurtosis (SE)	0.91 (0.99)	−1.22 (0.99)	0.15 (1.12)	−0.18 (1.12)		Previous combat experience	*F*_(1, 66)_ = 14.18	<0.001
Kolmogorov-Smirnov test^a^, *Z* (*p*)	0.99 (0.28)	0.56 (0.92)	0.78 (0.58)	0.93 (0.35)				

a *testing the normality of the distribution of the AAS score*.

b *Bonferoni corrected, critical p-value < 0.008*.

c *testing homogeneity of variances*.

*NC, Non-combatants; C, Combatants*.

We would like to qualitatively expound on the result that female combatants can be motivated by pleasure or enjoyment to perpetrate violence in a manner similar to male combatants by presenting a few quotations from the interviews. It should be noted that the following descriptions, while often consistent with international definitions of war crimes, are not inherently out of the ordinary when working in conflict settings. One of the female combatants reported: “When I tortured someone, I liked to do it slowly. Using my bare hands, I would scratch and pull off the penis. Sometimes it would take more than an hour.” Another shared: “I enjoyed most to burn civilians. I told them they would be safe in the building. But then I locked them in and set it on fire. I watched them through the window. I still get wet thinking about it.” And another reported: “I kept my prisoners in a hole outside, and I told them every night before I went to bed that I would kill them the next day. I did this for a year. That was the most fun.” Furthermore, many combatants asserted the perception that female combatants were more dangerous and cruel than males and that they would thereby, in the case of being taken prisoner, prefer male to female captors.

## Discussion

In this investigation, we interviewed male and female combatants and civilians in order to assess their perceptions of violent and aggressive behavior as quantified by AAS sum scores. There was no difference between male and female combatants in appetitive aggression, demonstrating that, in a context in which it is situationally appropriate to directly perpetrate violence against others and when males and females have perpetrated similar types of violent acts, both males and females are capable of experiencing aggression as fascinating or pleasurable. Furthermore, the significant difference in AAS sum scores between individuals with combat experience and those without further supports the conclusion that the development of appetitive aggression is related to the perpetration of violent acts. This finding highlights the dynamic nature of appetitive aggression that cannot be simply reduced to sex differences.

### Strengths and limitations

As mentioned above, at the time of the study, there was greater accessibility to former male combatants than female combatants because they were affiliated with separate organizations. The disparity in the numbers of in the former combatant and control groups was caused by the fact that we were not yet well-connected to the former female combatant organization and therefore did not have as much access as originally intended. While equal group sizes would have been preferred, we decided against artificially excluding participants.

Amongst the female combatants with whom we talked, we found on average high degrees of appetitive aggression. Selection effects presumably contribute to this phenomenon. The very process of acquiring a weapon to attain fighter status and then having subsequent success to be able to survive on the battlefield is a very challenging undertaking and signals high levels of toughness and determination. Due to obstacles established by cultural norms and a constant threat of abuse by both enemies and allies, presumably all female combatants have to adapt to a perilous environment in order to first be accepted by their comrades. This impression that the female combatants faced formidable obstacles from the very onset of joining armed forces was substantiated by numerous, unofficial participant reports that the initial training of female combatants usually included more life threatening situations and lasted longer in duration than that for the males. To subsequently be designated as fighters and then survive active combat are feats that likely requires even higher levels of grit and tenacity.

Furthermore, while we cannot ignore that sex differences in genetic predispositions may contribute to diverging appetitive aggression patterns in male and female populations, the finding that female combatants are even capable of experiencing similar levels of appetitive aggression as male combatants is nevertheless vital for updating current research efforts on the manifestation of appetitive aggression. Previous studies focused on male populations had findings that might also play a role in the development of appetitive aggression in females. For example a study with male combatants and former combatants in Burundi found that childhood maltreatment fosters appetitive aggression (Nandi et al., 2015). Another study identified appetitive aggression as a risk factor for current violent behavior (Crombach and Elbert, 2014) and several studies confirmed that appetitive aggression might be as protective factor against the development of PTSD in male combatants (Weierstall et al., 2011, 2012a).

### Directions for future research

In an effort to confirm the results of our present study and in light of the additional findings from other projects, we have already implemented an investigation with a larger sample of female combatants in Burundi exploring other factors potentially influencing appetitive aggression, such as bonding experiences and maltreatment during childhood. Moreover, we are interested to see how appetitive aggression might influence current risk behavior, the readiness to use violence in parenting and the successful reintegration of combatants into civilian life.

## Conclusion

Appetitive aggression is not an experience unique to males. In a context such as combat, both males and females are capable of experiencing the perception of aggressive acts as appealing and pleasurable, suggesting that the experience of appetitive aggression and even “combat high” is likely more related to the levels of violence perpetrated than to sex differences. Further research is needed to specify the female experience of appetitive aggression and provide further insight into the overall development and manifestation of appetitive aggression.

### Conflict of interest statement

The authors declare that the research was conducted in the absence of any commercial or financial relationships that could be construed as a potential conflict of interest.
