# Supplementary material for: Appetitive Aggression in Women: Comparing Male and Female War Combatants
Source: Front Psychol. 2016 Jan 5;6:1972. doi: 10.3389/fpsyg.2015.01972 (PMC4700207; doi:10.3389/fpsyg.2015.01972)
Supplement: Supplementary file 1 [file DataSheet1.docx]

Appendix

Table of Traumatic and Perpetrated Event Types

| No. | Type | Item | Yes | No |
| --- | --- | --- | --- | --- |
| 1 | Traumatic event | Have you experienced a natural disaster (flood, landslide, volcanic eruption, earthquake) where your own life was in danger? |  |  |
| 2 | Traumatic event | Have you experienced a life threatening fire or explosion? |  |  |
| 3 | Traumatic event | Have you experienced a serious accident (e.g. car accident, serious accident at work or home)? |  |  |
| 4 | Traumatic event | Have you witnessed a serious accident? |  |  |
| 5 | Traumatic event | Have you suffered from an immediately life threatening illness or injury? |  |  |
| 6 | Traumatic event | Has a close friend or family member had an immediately life threatening illness or injury? |  |  |
| 7 | Traumatic event | Have you been physically assaulted (e.g. been attacked, kicked, beaten up (includes beating with sticks) so severely that you feared for your life? |  |  |
| 8 | Traumatic event | Have you witnessed somebody being physically assaulted (e.g. being attacked, kicked beaten up (includes beatings with sticks)? |  |  |
| 9 | Violent offense | **Have you physically assaulted another person?** |  |  |
| 10 | Violent offense | **Have you made another person scream in pain?** |  |  |
| 11 | Violent offense | **Have you talked to others about experiences where you inflicted harm?** |  |  |
| 12 | Traumatic event | Have you been assaulted with a weapon (e.g. being shot, stabbed)? |  |  |
| 13 | Traumatic event | Have you witnessed somebody being assaulted with a weapon? |  |  |
| 14 | Violent offense | **Have you physically assaulted someone with a weapon?** |  |  |
| 15 | Violent offense | **Have you made another person bleed?** |  |  |
| 16 | Violent offense | **Have you made another person suffer from physical pain?** |  |  |
| 17 | Violent offense | **Have you mutilated another person?** |  |  |
| 18 | Traumatic event | Have you seen someone being killed? |  |  |
| 19 | Traumatic event | Have you witnessed a suicide/suicide attempt? |  |  |
| 20 | Violent offense | **Have you killed another person?** |  |  |
| 21 | Violent offense | **Have you killed another person when you had the choice?** |  |  |
| 22 | Violent offense | **Have you harmed another person who could not defend themselves?** |  |  |
| 23 | Traumatic event | Have you experienced a sexual assault (someone touched your private parts against your will, rape)? |  |  |
| 24 | Traumatic event | Have you witnessed a sexual assault? |  |  |
| 25 | Violent offense | **Have you sexually assaulted someone?** |  |  |
| 26 | Traumatic event | Have you witnessed dead bodies? |  |  |
| 27 | Traumatic event | Have you witnessed a massacre? |  |  |
| 28 | Violent offense | **Have you participated in a massacre?** |  |  |
| 29 | Traumatic event | Has someone threatened to kill you? |  |  |
| 30 | Violent offense | **Have you made another person beg for their life?** |  |  |
| 31 | Traumatic event | Have you been deprived of food as an adult? |  |  |
| 32 | Traumatic event | Have you defended yourself in a fight? |  |  |
| 33 | Violent offense | **Have you desecrated dead bodies?** |  |  |
